# The Ski Climate Index (SCI): fuzzification and a regional climate modeling application for Turkey

**DOI:** 10.1007/s00484-020-01991-0

**Published:** 2020-08-26

**Authors:** Osman Cenk Demiroglu, Mustafa Tufan Turp, Mehmet Levent Kurnaz, Bruno Abegg

**Affiliations:** 1 Department of Geography and Arctic Research Centre (ARCUM), Umeå University, 901 87 Umeå, Sweden; 2 Center for Climate Change and Policy Studies, Bogazici University, 34342 Istanbul, Turkey; 3 Department of Physics, Bogazici University, 34342 Istanbul, Turkey; 4 Institute for Systemic Management and Public Governance, University of St. Gallen, 9000 St. Gallen, Switzerland

**Keywords:** Ski Climate Index (SCI), Fuzzy logic, Ski tourism, Climate change, Regional climate modelling, Turkey

## Abstract

Climatology has increasingly become an important discipline for understanding tourism and recreation, especially in the era of contemporary climate change. Climate indices, in this respect, have been useful tools to yield the climatic attractiveness of tourism destinations as well as in understanding their altering suitability to various tourism types along with the changing climates. In this study, a major gap for a comprehensive climate index tailored for ski tourism is aimed to be fulfilled. For this purpose, initially the Ski Climate Index (SCI) is specified, based on fuzzy logic and as informed by literature and through extensive co-creation with the ski tourism industry experts, and applied to an emerging destination, Turkey, based on regional climate modeling projections. The index is designed as a combination of snow reliability and aesthetics and comfort facets, the latter of which includes sunshine, wind, and thermal comfort conditions. Results show that the Eastern Anatolia region is climatically the most suitable area for future development, taking account of the overriding effects of natural and technical snow reliability. Future research suggestions include the incorporation of more components into the index as well as technical recommendations to improve its application and validation.

## Introduction

Climatology has increasingly become an important discipline for understanding tourism and recreation (de Freitas 2003; Martínez-Ibarra and Gómez-Martín 2013), especially in the era of contemporary climate change (de Freitas 2017; Fang et al. 2018). Climate indices (e.g., Mieczkowski 1985; de Freitas et al. 2008; Scott et al. 2016), in this respect, have been useful tools to yield the climatic attractiveness of tourism destinations as well as in understanding their altering suitability to various tourism types along with the changing climates.

Climate indices can be briefly defined as indicators that summarize meteorological variables such as temperature, precipitation, humidity, pressure, and wind into categorized, spatiotemporal values that are significant for various physical and human systems. For many decades, tourism has also been a subject of these indices since Mieczkowski (1985) introduced the Tourism Climatic Index (TCI), which yields the climatic potential of tourism destinations based on total scores derived from summations where the climatic components have their own weighted contributions based on individual rating scales.

According to de Freitas et al. (2008) and Scott et al. (2016), climate indices for tourism should be theoretically sound, simple to calculate, easy to use and understand and empirically tested, and integrate the effects of all facets of climate, including those overriding effects of certain weather conditions up to a degree that may cancel out all other positive weather conditions. The use of the highest temporal resolution is also suggested in order to reflect the tourist behavior in a more realistic fashion than that of the TCI, which utilized monthly data as availability of daily or diurnal data was fairly limited in the 1980s. In this respect, contemporary indices such as the Climate Index for Tourism (CIT) (de Freitas et al. 2008) and the Holiday Climate Index (HCI) (Scott et al. 2016) have been developed to represent beach and urban destinations, respectively, claiming to overcome the limitations of the TCI, namely the subjectivity of the rating scales and the weighting schemes, lack of overriding effects, low temporal resolution, and the inadequacy of a one-size-fits-all formula for all tourism types. As stated by Dubois et al. (2016: 349), “the use of complex indexes such as the Tourism Comfort Indexes and the new generation of derived indexes is useful, all the more if these indexes are adapted to different forms of tourism.”

In the case of the highly weather-dependent ski tourism sector, which is regarded as the most studied type of tourism under a changing climate (Scott et al. 2012), the 100-day rule (Witmer 1986) of natural snow reliability (Abegg 1996), could be, albeit single faceted, perceived as the first indexing attempt to assess the climatic suitability of ski areas, resorts, and destinations and the relevant impacts of climate change on them. The rule indicates that climatic suitability (and economic viability) for ski areas/resorts/destinations is only possible when a minimum of 100 days/season of snow cover of a minimum 30-cm depth is present. It has been used widely over the past three decades with modifications such as the ability to be operational on peak periods such as the Christmas–New Year break (Scott et al. 2006) and the consideration of snowmaking, which has rapidly diffused among the global ski domain for both competition and climate change adaptation reasons, adding technical snow reliability to the conventional natural snow reliability assessments (Steiger and Mayer 2008). Thus, the snow reliability indicator has over the years evolved from a single rule into a mix of different criteria (Abegg et al. 2020)—yet still lacking the non-snow components of the climate system, which might have significant implications for ski tourism development as determined by the studies reviewed below.

In addition to the aforementioned snow reliability assessments, some studies have attempted to incorporate other climate variables for understanding ski area/resort/destination attractivity as informed by the literature (Yu et al. 2009a) and/or from the perspectives of suppliers (Berghammer and Schmude 2014; Li et al. 2016; Yang et al. 2017; Cai et al. 2019) and consumers (Rutty and Andrey 2014; Demiroglu et al. 2018). As a pioneering study, Yu et al. (2009a) applied the Modified Climate Index for Tourism (MCIT) (Yu et al. 2009b) to an Alaskan ski area. Here, combinations of the four sub-indices, namely perceived temperature (measured according to wind chill), wind speed, visibility, and present weather (as coded by the World Meteorological Organization), yielded overall unsuitable-marginal-ideal conditions. Among the thresholds on ideal conditions are a − 10 to 32 °F (− 23 to 0 °C) perceived temperature, a wind speed less than 13 mph (21 km/h), and visibility more than 4 km. Following a literature review and interviewing ski industry stakeholders in the Alps, Berghammer and Schmude (2014) determined the climatic components and thresholds of an Optimal Ski Day (OSD) as no precipitation, a temperature between − 5 and 5 °C, a sunshine duration of minimum 5 h, a wind speed of maximum 36 km/h, and a minimum snow cover in the surroundings of the slopes, in addition to the commonly referred criterion for a minimum of 30 cm snow depth to make skiing possible, for a comfortable and aesthetic skiing experience. In order for a ski day to be classified as an OSD, all these six climatic conditions should hold true at the same time for the whole ski area on a weekend or holiday, maximizing attractivity and revenues. Although the OSD approach enhances the snow reliability indicator with other ski-specific climate indicators, it is considered that it may not serve as a full index as it weighs all of its components equally and imposes a coexistence rule on them—the latter binary condition meaning that a ski day can only be optimal when all components reach their ideal values and that any minor deviation from the ideal range for one single component will result in a non-optimal ski day. The OSD was later applied to a German ski area to investigate how projected climatic changes, along with demographic trends, could affect the visitation patterns (Witting and Schmude 2019). Similar to the OSD concept, Demiroglu et al. (2018) determined the Ideal Summer Ski Day as one that takes place on a weekend or holiday in May or June, when there is no wind, no clouds, a temperature between 10 and 20 °C, wet or corn quality of snow and a snow depth above 1 m. This indicator was based on consumer surveys rather than supplier interviews, but again without any prioritization among its components and only limited to the summer skiing context in Norway. Another consumer survey by Rutty and Andrey (2014) in Ontario, Canada, pointed out (freezing) rain occurrence and wind chill temperature as the most important weather attributes in ski trip decisions in Ontario, Canada.

In China, where ski tourism is facing a rapid growth (Wu and Qinghua 2018), Li et al. (2016), based on expert judgment, came a step closer to building a full climate index tailored for ski tourism by rating and weighting snow depth, snowfall duration, snow quality, lowest temperature, wind characteristics, and annual precipitation to assess the development conditions of five sample ski resorts in the north of the country. Although the interrelationships and the implications of these conditions were not made clear enough in the study, it may have formed a basis for building a comprehensive, multifaceted climate index for ski tourism. Likewise, Yang et al. (2017) developed another comprehensive index, informed by experts and scholars, where winter temperature as well as the spatiotemporal distribution of snow contributed as major components of an overlay analysis. Recently, Cai et al. (2019), departing from the *Meteorological Index of Skiing* developed by the China Meteorological Administration (2017), proposed the Meteorological Suitability Index (MSI), which takes account of wind and temperature conditions, and the Snow Abundance Index (SAI), based on snow thickness and duration, and applied them for Northeast China by coupling through the Copula function.

In this study, the aim is to contribute to the efforts on developing a comprehensive climate index for ski tourism development by taking into account additional non-snow climatic components in a relatively flexible way. In this respect, the Ski Climate Index (SCI) is initially specified as informed from literature and through extensive co-creation with ski tourism industry experts in Turkey, where an intensified growth of the sector has been observed over the past two decades and the authorities project the number of domestic skiers to grow into 4 million and the ski resort development investments to add up to €48.5 billion by the 2020s (Hudson and Hudson 2015; Göymen et al. 2017). The SCI is applied to assess the future ski tourism climatology of Turkey and to distinguish those regions, at a destination scale, with comparative climatic advantages, based on projections of the regional climate model RegCM4 (Giorgi et al. 2015). A comparison is also made by interpreting the results with regards to the distribution of existing and proposed ski areas.

## Materials and methods

The multi-method approach of this study includes the definition and construction of the SCI in terms of its facets and variables and employing a regional climate model to project the values that will feed the SCI for an application on Turkey. Related data and techniques are further elaborated below.

### Specification of the SCI

The specification of the SCI is initially based on varied consultations with ski industry experts through the *Turkish Ski Tourism Forum* (Demiroglu 2015), the *SECTEUR: Sector Engagement for the Copernicus Climate Change Service: Translating European User Requirements* (ECMWF 2015), the *Technical Assistance for the Development of a Winter Tourism Corridor in Erzurum, Erzincan and Kars* (Turkish Ministry of Culture and Tourism 2017), and the *Overall Assessment of the Turkish Mountains and Inventory of the Best Ski Sites* (Compagnie des Alpes 2017) projects as well as the findings of the above-cited literature. Accordingly, the index facets as well as their sub-indices and relatedness, are identified and overlay options are suggested. The main facets of the *SCI* are determined as snow reliability (SR) and those non-snow components related to aesthetics and comfort (AC), such as sunshine, wind, temperature, and humidity.

In order to deal with the relative inflexibility arising from conventional utilization of discrete scaling, weighing and overlay techniques in standardization and construction of tourism climate indices, we opt for assigning fuzzy memberships to the crisp, i.e., the observed, values per component and employing fuzzy operators (see next paragraph) of overlay within and between the facets of the *SCI*. As Mitchell (2012, p. 129) argues, in traditional set theory, one follows binary reasoning and decides whether a crisp value meets a certain requirement or not (is a member of the set or not), whereas in fuzzy logic, the main concern is the likelihood, i.e., the fuzzy membership value, that a crisp value is a member of the set, following a continuous scale from 0 to 1 rather than a strictly binary decision. Such tools of fuzzy logic are becoming more common in tourism climatology (Olya and Alipour 2015; Cai et al. 2019), where indexing attempts involve highly subjective inputs with usually non-discrete ranges. As a first step of the fuzzy membership assignment, i.e., the fuzzification process, the input crisp values are given a membership likelihood value based on the scale of 0 to 1 where different relationship functions (Mitchell 2012; Esri 2017) such as LINEAR, NEAR, GAUSSIAN, SMALL, LARGE, MS (stands for mean and standard deviation) SMALL and MS LARGE are in play for continuous data. Manual membership assignment to categorical data is also applicable, yet less relevant as the conventional reclassification techniques may also suffice. The LINEAR function transforms the inputs on a slope with options to set optimal values as thresholds to exclude or give full membership to certain values of the original set. The GAUSSIAN, NEAR, SMALL and LARGE functions assign the highest membership values to either the specified midpoints or the minimums/maximums with an optional spread parameter to set the rate of change, with the main difference between GAUSSIAN and NEAR being a narrower spread around the midpoint for the GAUSSIAN. MS SMALL and MS LARGE, on the other hand, resemble SMALL and LARGE functions, yet here MS SMALL sets the input values less than the mean to a full membership whereas MS LARGE transforms them to non-members. The fuzzification process also enables the use of the so-called hedge factor that helps to account for the (un)certainty of qualitative expert opinions under the VERY and the SOMEWHAT parameters which favor extreme and moderate fuzzy memberships for the observed values, respectively (Raines et al. 2010). For example, compared to the results of a LARGE fuzzification process, a set of observations with a VERY LARGE function would end up having higher fuzzy membership values for the high crisp values. In other words, higher observed values would be closer to the maximum fuzzy membership value of 1.

The overlay techniques in fuzzy logic (Mitchell 2012) are also diverse compared with for instance weighted sum or weighted overlay approaches, as one can combine different fuzzified layers with various mathematical or logical operators. In essence, the PRODUCT operator tends to be decreasive and honors suitability to locations where all inputs perform well. It is best used for finding the optimal locations. The SUM operator, which indeed is not literally a summation but a complement of the product of all inputs’ complements, has an increasive tendency to highlight all the potentially suitable locations. The more sophisticated GAMMA operator combines the PRODUCT and the SUM operators, where one can modify a gamma factor (*G*) value to fine-tune the model increasively or decreasively according to whether the aim is toward the optimal or the potential solutions. The logical operators AND and OR, on the other hand, strictly exclude and include certain inputs, respectively, by defining an intersection set (∩) or a union set (∪) among them. They could be preferred over the mathematical operators to avoid any overestimation should any input sets be potentially correlated.

As confirmed by ski tourism stakeholders, snow is the most essential climatic factor, making SR the most important facet, which is a function of depth and duration of natural and technical snow. This facet is primarily measured by the climatic average (30 years) of the number of days with sufficient snow depth for downhill skiing during the winter tourism season from December 1 to March 31 (DJFM). Here, April is excluded, despite its snow accumulation potential, due to the likely backyard behavior of demand, that is, the effects of one’s home weather conditions on her/his ski trip decision (Hamilton et al. 2007), which could reduce visitation down to as low as 0.5% of the total visits as in the case of Turkey (Demiroglu 2016). Regarding the skiable snow depth, a minimum requirement is identified as 30 cm for a prepared ski slope (Witmer 1986; Abegg et al. 2007), where grooming operations compress the density of snow to around 450 kg/m^3^ (Fauve et al. 2002 in Mayer and Steiger 2013: 176). In other words, snow cover with a snow-water equivalent (SWE) of 135 kg/m^2^ could satisfy the minimal conditions for SR, as SWE is a product of snow density and snow depth. For standardization purposes for this first sub-index of the SR facet, namely natural snow reliability (NSR), the seasonal (DJFM) average number of naturally skiable days in a climatic range of 30 years is fuzzified by the LARGE algorithm, where observation values above a midpoint of 100 days are honored with higher (more than 0.5) membership values.

As SR is also a matter of technical efforts besides the natural features, snowmaking (SM) appears to be another component to be considered within the SR facet. As noted by experts and operators, as well as the literature (see Demiroglu et al. 2016 for a local example), in order for the common snowmaking systems to produce high quality, less dense snow, cold ambient temperatures are required, depending on relative humidity. In areas with higher relative humidity, colder surface temperatures will be needed, and vice versa, therefore, here the wet-bulb temperature (WBT) acts as a useful variable for a correct assessment of snowmaking activities. In this study, a highly conservative maximum threshold of − 7 °C WBT is applied in order to selectively distinguish regions in terms of their both quantitative and qualitative snowmaking capacities (Demiroglu et al. 2016). In the light of these parameters, the total snowmaking component is measured as the number of total hours that meet the aforementioned threshold during the pre- and the actual ski season months of November to March (NDJFM), as technical efforts are usually in effect prior to the season as well for base layer formation. Finally, a LINEAR fuzzification that favors higher number of hours is realized.

A final component of the SR facet is the condition of being operational on and around the official New Year’s Day holiday when generally the demand peaks and the prices are maximized. In Turkey, the stakeholders state that as much as 5 to 15% of the total revenues for the whole season could be generated during the New Year’s Holiday, depending on its extension from or to a weekend. Moreover, as the policymakers aim for the internationalization of Turkish ski tourism (Göymen et al. 2017), thus target for an extended holiday calendar of the incoming markets, it is still very crucial for (potential) Turkish ski businesses to have sufficient snow conditions for skiing before the New Year’s Day. This condition (NY) is added to the index in both natural and technical terms. Regarding natural snow reliability (NY-NSR), the probability of having an SWE over 135 kg/m^2^ on every last day of December within a climatic range of 30 years is computed. As for technical SR (NY-SM), it is reported that the average snowmaking systems could prepare a base layer in 72 hours (Mayer and Steiger 2013: 175). Therefore here, the probability of achieving this requirement, based on the maximum − 7 °C WBT threshold, a more conservative threshold to the − 5 °C value identified in Mayer and Steiger’s (2013) study, during the months of November and December (ND) is calculated. For simplification reasons regarding the large regional extent of this study, this approach does not account for a combined effect of natural and technical potentials, but rather opts for the best of the two likelihoods, based on the logical operator OR. While the base-layer snowmaking season of ND may seem problematic as it may be subject to warming snowmelt periods in its relatively long range, November is nonetheless included since, in the case of Turkey, the experts indicate the presence of competition for season-opening on December 1 to 15. Fuzzification for NY-NSR follows an algorithm favoring VERY LARGE crisp values to emphasize the likelihood of being operational throughout the whole climatic range, while NY-SM is modeled on a SOMEWHAT LINEAR trend with a minimum threshold below 72 h.

The final equation (1) for the SR facet is determined by an exclusive fuzzy overlay with the logical operator AND in order to ensure that locations meet all three criteria at their bests. Such design also helps considering overriding effects of the lack of each condition, to an annihilating degree. Using mathematical operators such as SUM and PRODUCT is avoided here as snow and temperature, as well as the natural and the technical sub-indices defined under different terms, are expected to be highly correlated (Mitchell 2012: 160).

1 $$ SR= NSR\cap SM\cap \left( NY- NSR\cup NY- SM\right) $$

Sub-indices of the facet for aesthetics and comfort (AC) are also measured with respect to their likelihoods of occurrence. In the case of Turkey, the ideal threshold for the SUNSHINE (SS) duration per day, which takes account of the cloud cover, is set as a minimum of 6 hours—higher than the Alpine threshold of 5 h/day (Berghammer and Schmude 2014), given the extended daylight of southerly latitudes in the Northern Hemisphere. Regarding wind conditions (WC), a 40-km/h daily maximum speed is set as the threshold, as it denotes not only the maximum tolerated windiness by skiers but also a safety limit above which aerial lift operations may be suspended, according to the experts. Last but not least, the thermal comfort (TC) range, formerly defined in the OSD as a temperature between − 5 and 5 °C (Berghammer and Schmude 2014), which is based on a slightly cool perception of the physiological equivalent temperature (PET) (Matzarakis et al. 1999), is redefined within a WBT range of − 7 to 2 °C for 9 am to 6 pm every day, accounting for the effects of relative humidity sub-diurnally only during the actual ski time. In other words, TC’s WBT range is equivalent to OSD’s dry-bulb temperature range corrected (Stull 2011) by relative humidity of 71%, a reference value representative of Turkey’s average relative humidity for the ski season (DJFM) during the 1970–2019 period (Turkish State Meteorological Service 2020). All three sub-indices are fuzzified in a way to favor the LARGE number of days that meet their respective thresholds. The final equation (2) of the AC facet is a fuzzy SUM of these three components as they are expected to form a synergy where their combined effect is higher than their individual effects (Mitchell 2012: 158). Alternatively, a fuzzy PRODUCT could also be employed for the overlay, despite its overall decreasive nature, in order to account for the overriding effects of adverse winds to an annihilating degree.

2 $$ AC=1-\left(1- SS\right)\ast \left(1- WC\right)\ast \left(1- TC\right) $$

Taking all the parameters above into consideration and the practical use of the GAMMA operator for an overall combination of fuzzy overlays (Mitchell 2012: 159f), the generic formula (3) for the SCI is specified as:

3 $$ SCI={\left(1-\left(1- SR\right)\ast \left(1- AC\right)\right)}^G\ast {\left( SR\ast AC\right)}^{1-G} $$

where SCI is the total climatic suitability score for a (potential) ski area, resort or destination, depending on the spatial scale in scope, SR and AC stand for the facets of snow reliability and aesthetics and comfort; respectively, and G is the GAMMA factor that has a value range of 0 to 1 to have control over being decreasive or increasive. In this study, a gamma factor of 0.9 is applied in order to gain more from a SUM effect and highlight as many suitable areas as possible, while still maintaining the annihilating effects of any lack of SR through the final multiplication of the GAMMA controlled SUM and PRODUCT results. However, it should also be noted that some degree of correlation may be present between the SR and the AC facets due to the uses of WBT value in TC, NY-SM, and TSR computations. The final index score is yielded within a range of 0.00 to 1.00, as with all other fuzzy memberships. The higher the final score, the better the suitability will be. The architecture of the SCI is summarized in Table 1.

**Table 1:** Definitions, fuzzification, and overlay of the Ski Climate Index (SCI) facets and sub-indices

Facet	Overlay	Sub-index	Definition	Fuzzification
Snow reliability (SR)	AND	Natural snow reliability (NSR)	Seasonal (DJFM) average number of days when SWE is larger than 135 kg/m^2^ in a 30-year range	LARGE (midpoint: 100, spread: 10, hedge: VERY)
		Snowmaking (SM)	Pre- and actual seasonal (NDJFM) average number of hours when WBT is < − 7 °C in a 30-year range	LINEAR (hedge: none)
		New Year’s Day Opening (NY)	Ratio of number of days when SWE is larger than 135 kg/m^2^ on every Dec. 31 in a 30-year range (NY-NSR) OR Pre-seasonal (ND) average number of hours when WBT is < − 7 °C in a 30-year range (NY-SM)	LARGE (midpoint: 0.5, spread: 8, hedge: VERY) LINEAR (minimum: 71, hedge: SOMEWHAT)
Aesthetics and comfort (AC)	SUM	Sunshine duration (SS)	Seasonal (DJFM) average number of days when the sunshine duration is more than 6 h in a 30-year range	LARGE (spread: 5, hedge: none)
		Wind conditions (WC)	Seasonal (DJFM) average number of days when the top wind speed is less than 40 km/h in a 30-year range	LARGE (spread: 5, hedge: none)
		Thermal comfort (TC)	Seasonal (DJFM) average number of days when WBT is between − 7 and 2 °C from 9 am to 6 pm in a 30-year range	LARGE (spread: 5, hedge: none)

### A regional climate modeling and geographical information system–based application of the SCI for Turkey

Turkey, for several years, has been among the top 10 countries of the world in terms of international visitor arrivals (World Tourism Organization 2019), owing to its competitive offers in the beach, cultural, and health tourism (Duman and Kozak 2010; Ceti and Unluonen 2020). In line with its tourism diversification policies and to foster regional development, the country announced a macro strategy in 2014 to identify and invest in new ski areas in the next 12 years (Hudson and Hudson 2015; Göymen et al. 2017). In this respect, application of the SCI is seen as a good opportunity for both as a major suitability determinant, besides other factors such as topography, land use, and accessibility, and to compare the index itself based on the locations of existing and proposed ski areas (Göymen et al. 2017).

In order to calculate fuzzy membership scores for facets and the sub-indices of the SCI, values on certain climatic variables such as snow-water equivalent, wet-bulb temperature, maximum wind speed, and sunshine duration need to be obtained or computed. As only a very limited number of active meteorological stations in Turkey have long-term observations of the snowy terrain above 2000 m and the future concerns are of importance for site selection toward ski tourism development, we employ a regional climate model, RegCM 4.4 (Giorgi et al. 2015), in order to compute reference and future projections of the four variables, on a grid basis, to be used as inputs to the SCI. At the first phase, a dynamical and double-nested downscaling of the Earth system model MPI-ESM-MR (Max Planck Institut für Meteorologie 2012), which is a commonly utilized model with exact Gregorian calendar settings including the intercalary years, to a resolution of 0.44° by 0.44° and further down to 0.08° by 0.08° is realized regarding the Turkish domain. Regarding parametrization, Biosphere and Atmosphere Transfer Scheme (BATS) (Dickinson et al. 1993) for the land-surface scheme, Holtslag scheme (Holtslag et al. 1990) for the planetary boundary layer scheme, and Grell (1993) scheme with the Fritsch and Chappell (1980) type closure for the convection scheme are all included in the model, as previously applied by Turp et al. (2014). The temporal ranges are set to 1971–2000 for the reference period and 2021–2050 for the future. Future projections are carried out along the RCP 8.5 scenario, which stabilizes radiative forcing at 8.5 W/m^2^ in a future world characterized by high energy demand and absence of climate change policies (Riahi et al. 2011). RCP 8.5 is the most pessimistic, business-as-usual trajectory utilized by the Intergovernmental Panel on Climate Change (IPCC) and is preferred in this study to assess the ski tourism development potential of Turkey by reflecting on the worst conditions to come.

As a result of the dynamical downscaling process, grid point values on the four variables are projected with a spatial resolution of 0.08° by 0.08° (approximately 10 km by 10 km) and a temporal resolution of 3 h under historical and future datasets. The critical SWE values are computed for each grid by considering numerous parameters such as land type, soil information, net surface heating, and soil or snow heat capacity since the land surface processes are coupled to the RegCM via the Biosphere-Atmosphere Transfer Scheme (Dickinson et al. 1993). This relationship could be formulated as:

4 $$ \frac{\partial SWE}{\partial t}={P}_s-{S}_m-{F}_q $$

where SWE is the snow-water equivalent, *t* is time, *P*_*s*_ is the rate of snow precipitation, *S*_*m*_ is the rate of snowmelt, and *F*_*q*_ is the rate of sublimation. WBT values are computed as a function of near-surface temperature and near-surface relative humidity variables (Stull 2011). The relatively high temporal resolution of RegCM 4.4 has enabled us to distinguish the sub-index values sub-diurnally for the TC sub-index, which would otherwise have been under-/over-estimated by a daily resolution.

**Fig. 1:**
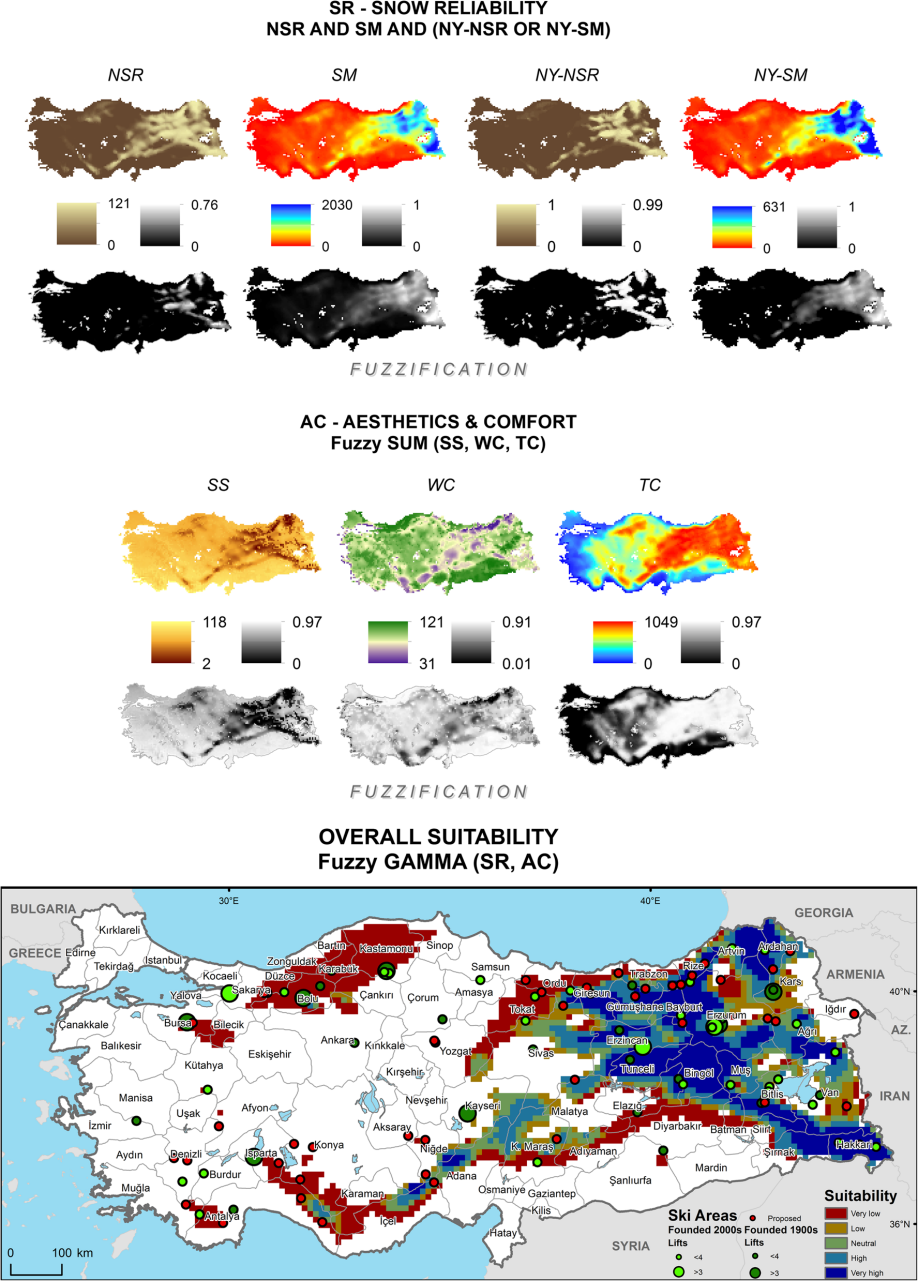
Historical (1971–2000) SCI projections over the Turkish Terrain

**Fig. 2:**
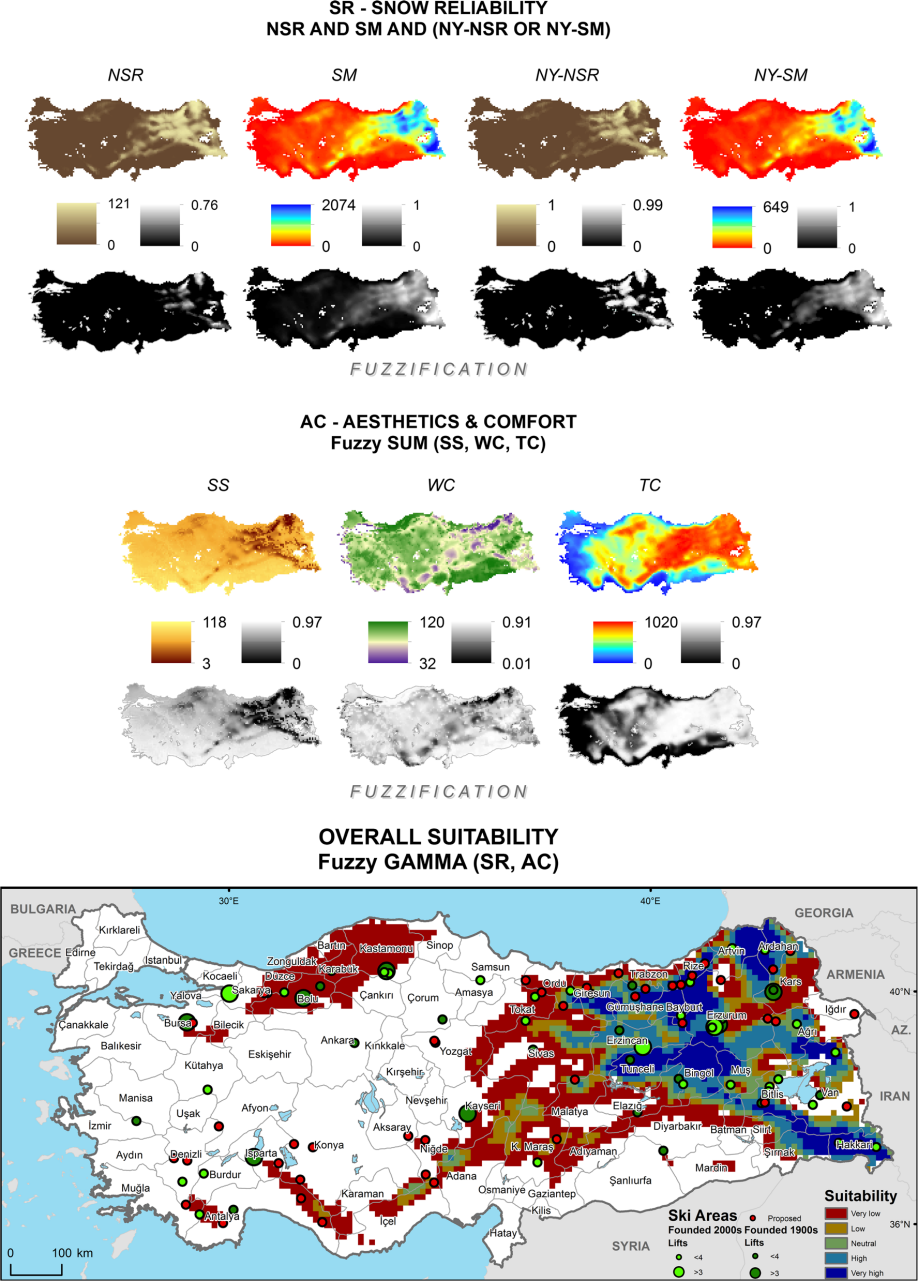
Future (2021–2050, RCP 8.5) SCI projections over the Turkish Terrain

Following the computation of crisp values for the SCI sub-indices in the CDO (Climate Data Operators) software for both temporal ranges, the point data are rasterized in the ArcGIS software, based on their actual spatial resolution, for further fuzzification processes where the observed values are assigned with fuzzy memberships and hierarchically overlaid according to the parameters summarized in Table 1. The study does not attempt any further interpolation to improve resolution and is limited to highlighting those destinations that consist of mostly homogeneously extending, high-altitude terrains (with base elevations above 1300 m, depending on latitude, aspect, continentality, etc. in the case of Turkey). It should also be noted that the projected data are not bias-corrected due to lack of reanalysis data on all SCI variables as well as the ongoing debates on the use of bias correction such as lack of a satisfactory physical justification (Ehret et al. 2012), the inflation issue especially regarding quantile mapping (Maraun 2013) and the essential need for long time series of observations (Maraun and Widmann 2018, p. 172). For these reasons, historical (Fig. 1) and future (Fig. 2) SCI scores, under a classification of five equal intervals that increase with break values of 0.2, are visualized according to this relatively coarse resolution. Zonal means per province—where the highest subnational public authorities of destination development are present—are also provided (Table 2) to interpret the results more comparatively at a larger regional scale. In order to introduce the case country more to the non-native reader, Table 2 presents also information on the relevant physical and human features of the provinces, including results of the most recent Socioeconomic Development Index (SEDI) assessment (based on demographic, economic, educational, health, competitive and innovative capacity, accessibility and life quality indicators), where a final categorization from 1 to 6 represents the most developed to the least developed, respectively (Turkish Ministry of Development 2013).

**Table 2:**
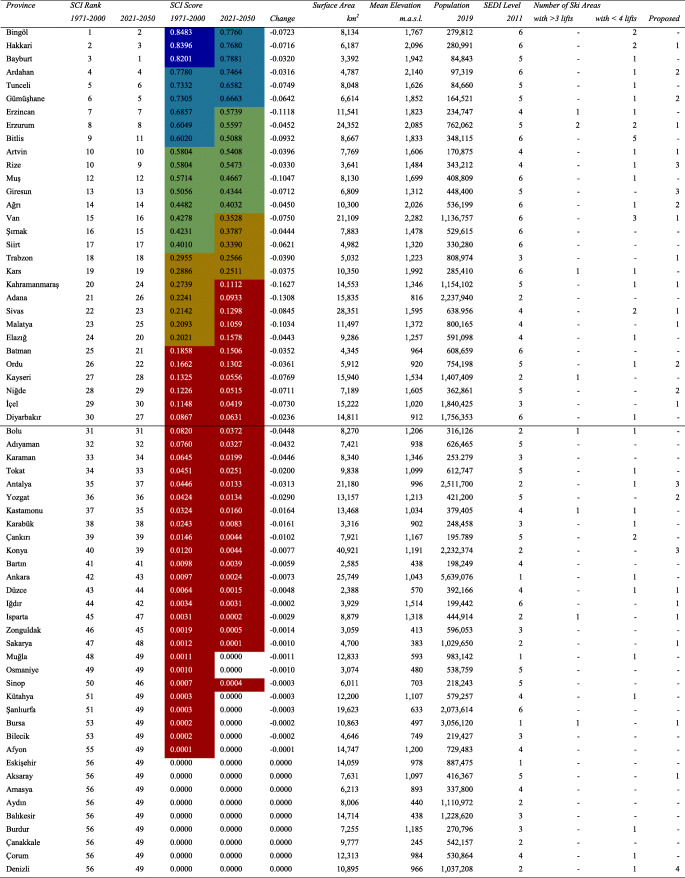

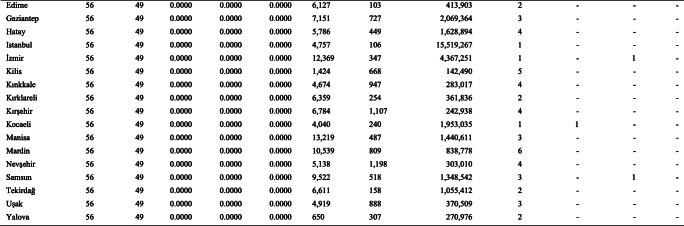
Summary of zonal SCI means per province in Turkey

## Results

The results section is organized into, first, an overall examination of the spatial distribution and the future temporal shift of the SCI and its sub-index scores (Figs. 1 and 2), then individual inspections of the provincial SCI performances over time (Table 2), and finally a comparison of the projected SCI scores across existing and proposed ski areas in Turkey (Göymen et al. 2017). The results are further discussed in the next section in terms of their limitations and future research suggestions.

### Spatiotemporal distribution of SCI scores over the Turkish terrain

RegCM 4.4 based historical (1971–2000) projections of *SCI* scores over Turkey (Fig. 1) highlight a very high climatic suitability for ski tourism development partly on the Central Taurus Range along the İçel-Konya (Bolkar Mountains) and the Adana-Niğde (Aladağlar Range) provincial borders and mainly within the provinces of Ordu, Giresun, Gümüşhane, Bayburt, Trabzon, Rize, and Artvin in the Eastern Black Sea region and Sivas, Erzincan, Erzurum, Ağrı, Muş, Bingöl, Tunceli, Elazığ, Bitlis, Van, Hakkari, Şırnak, Siirt, Batman, and Diyarbakır in the Eastern and Southeastern Anatolia regions. Along the immediate concentric circles of these regions, as well as the Tahtalı and the Binboğa ranges at the intersection of Adana-Kahramanmaraş-Sivas-Kayseri provinces on the easternmost limits of the Central Taurus, high suitability zones are projected. Such zones at smaller extents are found on the Köroğlu Mountains in Bolu and around the Geyik Range of the Western Taurus along the Antalya-Konya border. Climatically potential ski zones with medium, low, and very low suitability are concentrated on the Southeastern Taurus, on an arc from the lower maritime Eastern Black Sea Mountains to Tokat and Yozgat provinces, most of the Western Black Sea, the westernmost edge of Western Taurus in Antalya and Muğla, and Mt. Uludağ in Bursa. Besides, single volcanoes such as Muratdağı in Kütahya, Erciyes in Kayseri and Karacadağ on the Şanlıurfa-Diyarbakır border show partial climatic suitability for ski tourism development. While overall and New Year’s natural snow reliability sub-indices, as well as thermal comfort, have relatively homogeneous positive effects over these mountainous regions, snowmaking sub-indices, especially before New Year’s Day, favor Eastern Anatolia. Adverse winds (WC) and cloud formation (SS), on the other hand, reduce the overall performance, especially in the Eastern Black Sea region.

Turkey’s near future (2021–2050) climatological potential in terms of ski tourism development is projected to shrink (Fig. 2). In the western half, the least suitable regions lose their extents and those superior areas, such as on the Köroğlu Mountains (Bolu) and the Central and Western Taurus, shift down at least one lower suitability class. A similar trend is also projected for the eastern half, yet here high summits of Trabzon and Rize (Kaçkar Mountains), inner highlands of Giresun, parts of Artvin, Ardahan, Erzurum, Erzincan, Tunceli, Muş, Bitlis, Şırnak, and north of Lake Van, and most parts of Hakkari, Bayburt, and Bingöl remain strong in terms of their climatic suitability for ski tourism. In addition to a slightly weakening natural snow reliability, reductions in snowmaking capacities before the New Year’s Day appear to be the major determinants of change.

### SCI performances of Turkish provinces

Table 2 summarizes zonal SCI means per province in Turkey, taking account of unsuitable areas (white in Figs. 1 and 2) as well. The results may have been portrayed differently should only the mountains be in scope, whereas an overall provincial zoning approach favors those administrative regions with a highly mountainous and/or snowy character as well. Such climatic prominence, among other physical and human factors, of provinces provides them with the opportunity to be prioritized for professional ski destination development, which was the main vision of the Turkish Ski Federation during the implementation of the Overall Assessment of the Turkish Mountains and Inventory of the Best Ski Sites project (Compagnie des Alpes 2017). Out of the 81 provinces, 55 and 48 reflect at least some climatic suitability for downhill skiing for the historical and future periods, respectively. While future SCI performance is annihilated in seven provinces, the rest also pursues negative changes in their mean values, without any exception, and some become reclassified in terms of their suitability. In the top three performers, for instance, the ranking changes with historically third place Bayburt taking the lead from Hakkari in the future, while all three are projected to perform slightly below a very high suitability threshold in the future 2021–2050 period, based on the high emissions, business as usual RCP 8.5 trajectory.

### Comparison of the SCI results with observed conditions

**Fig. 3:**
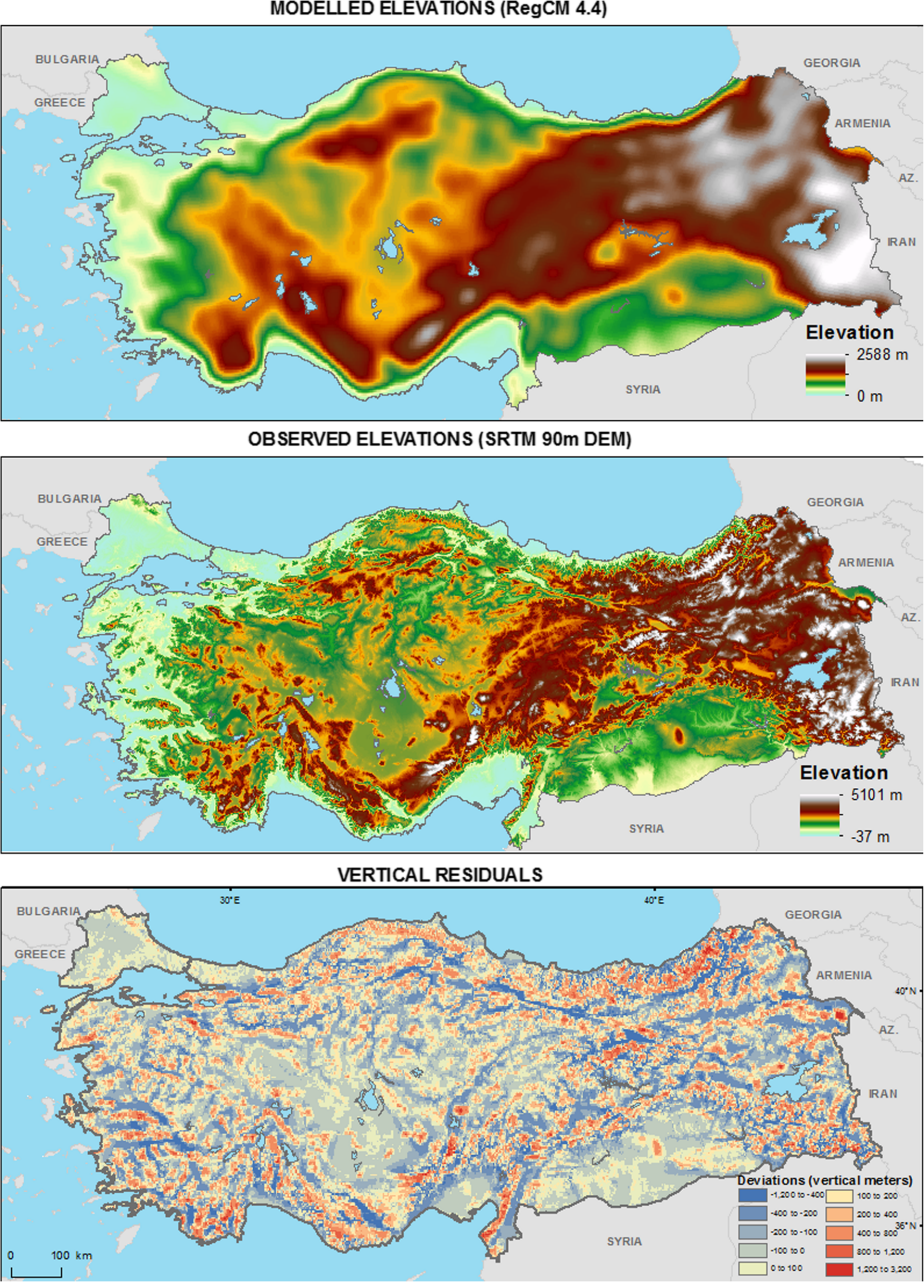
Vertical residuals between modeled and observed elevations

In order to test the performance of the SCI in terms of its underpinning information and the consequent design, the projected historical results can be compared with the observed distribution of ski tourism supply (Demiroglu 2008) in Turkey (Fig. 1). While an overall visual inspection shows that the SCI covers mostly the existing and the proposed ski area locations, there are a few notable exceptions. For instance, one new and one large old ski resort in Kocaeli and Bursa provinces lie within no suitability and very low suitability zones, respectively. This mismatch of ski area locations with suitable zones could be attributable to the fact that ski area/resort site selection is based on not only climatic but also topography, land cover and use, and the everchanging socioeconomic and sociopolitical factors such as cultural interests, income levels, manmade accessibility, and (perceived) security. (Li et al. 2016; Deng et al. 2019; Demiroglu in review), as well as the spatial resolution of the model. Reconsidering these two examples, the very high accessibility of these two resorts from large urban markets such as Istanbul and Bursa could justify their locations even if their relative climatic potentials were not strong. Regarding the latter attribution, Fig. 3 displays how the model topography deviates from an ultra-high resolution (90 m) digital elevation data (Consortium for Spatial Information 2018) of the actual topography, with a certain smoothing-out effect. The residuals mostly remain in a range of ± 400 m vertically, but increase up to as high as − 3200 to + 1240 m, especially around immediate peaks. Running spatial autocorrelation on the residuals returns a significant (*p* < 0.001) Global Moran’s Index value of 0.52, indicating that the extreme deviations are spatially clustered (Esri 2020). Therefore, it should be noted that the application of the SCI in this study aims for pointing out those destinations that consist of widely extending, high-altitude terrains. Thus, it is limited to the larger ski tourism destination scale rather than the more detailed ski area or resort scale.

## Discussion and conclusions

This study aimed at establishing an expert informed climate index tailored for ski tourism and its initial application on an emerging destination, Turkey, based on historical and future projections. Departing from consensus on the prioritization of relevant weather conditions and their combinations, coupled with frequent uncertainties on their specific magnitudes, the Ski Climate Index (SCI) was suggested by means of fuzzy logic, where the SR facet had the dominating role on the overall performance and the AC facet, based on sunshine, wind, and thermal comfort conditions contributed to further refinement. The results revealed that all other physical and human factors being equal, the climatologically primary zones for ski tourism development in Turkey lie in Eastern Anatolia, currently almost underutilized, and posing an opportunity for the future of the region. In this respect, more development proposals may need to be considered especially in the provinces of Hakkari, Bayburt, and Bingöl, while those projects planned in the Central Taurus and the Eastern Black Sea regions may need to be reconsidered due to their deteriorating snow conditions and/or exposure to strong breezes and high cloud cover. In any case, it is imperative that the considerations on SCI performance for future projects be based on projections, rather than the conventional observation-based decision-making, to account for the impacts of climate change. Then, however, the projections need to be based on the outputs of as many climate models and representative concentration pathways as possible, which is a major limitation of this study. Employment of non-hydrostatic regional climate models would also increase the quality of outputs with their ultra-high resolutions that account for topographic diversities. Otherwise, further statistical downscaling techniques (see Steiger 2010; Spandre et al. 2019) could also be employed and compared with this study. Last but not least, it is better to rely on the differences, rather than the absolute values, of reference, and future period climate projections. However, in order to use such deviation values, one needs proper observation data, which is not available in Turkey as the critical high-altitude snow depth measurements are very limited.

The novel index introduced in this study is meant to be of use for not only emerging but also established destinations as their attractiveness assessments should be based on all vital climatic (and non-climatic) factors besides snow conditions. For example, an existing ski area that is projected to get more snowfall in the future, but also more stormy days, may not be as viable as one may interpret on snow data only. Unlike the natural and/or snow-only indices (e.g., Abegg 1996; Scott et al. 2006; Steiger and Mayer 2008), SCI can capture that, as it did for Turkey’s Eastern Black Sea region along the Kackar mountains. It also fills in the gap posed by indices that have very limited or no coverage of snow conditions (Yu et al. 2009a, 2009b). Moreover, the SCI provides maximum flexibility in mixing its components, contrary to other indices that yield only the ideal climatic results (e.g., Berghammer and Schmude 2014; Demiroglu et al. 2018). In doing so, it does not limit its overlaying capacity with discrete ratings and weights (Li et al. 2016; Yang et al. 2017) but offers the power of fuzzy logic to deal with many uncertainties associated with ski tourism climatology.

Future studies on the SCI and its applications could focus on further iterations with the field experts, as well as the growing econometric studies on the demand side (see Mayer et al. 2018 for a review), to incorporate other components such as visibility, defined in terms of the number of fog events. Indeed, a domestic tour operator in Turkey has already introduced a mobile application to interact with its consumers on a real-time basis and alerts them based on a scale of 1 to 10, measured by inputs on snow depth, cloud cover, and windiness. Such sensitivities also highlight the importance of mixed effects, such as bluebird days, when a night’s, preferably low density (powder), snowfall is followed by a clear sky day with no winds. Contrary to these ideal wild snow conditions, the experts note that hard-packed manmade snow is also demanded by certain market segments, especially by alpine racers, therefore the timing of warm spells needs also be well-known to the operators during the base layer formation periods. Regarding thermal comfort, the wind-chill factor, in addition to the humidity effects, needs to be included to provide alternative measurements to apparent temperatures. The experts also note the importance of the backyard effects, indicating that urban snowfall significantly influences ski trip decisions, as previous empirical studies put forward (Hamilton et al. 2007). In this respect, an additional component could be included in the design of the SCI, by relating to the snowfall days in the major source markets. Last but not least, the calendar effects, which was only demonstrated by the peak New Year’s Day period, could be enriched according to the geographical scope of the index. In the case of Turkey, the two official religious holidays *Ramazan Bayramı* (Eid al-Fitr) and *Kurban Bayramı* (Eid al-Adha), which are dated according to the Islamic Lunar Calendar and will in the near future fall into the ski seasons, are potentially significant periods of high demand for domestic as well as the emerging Western Asian markets. The 2-week long school break in February is another period to be considered, especially as a spatiotemporal dispersion of this period is sometimes on the policy agenda (Demiroglu 2015). It is also possible to envisage the New Year’s Day period within a more extended perspective as both the western and the eastern Christmas breaks are relevant to Turkey’s future targeted ski markets, such as the UK and the Netherlands, and the conventional markets such as Russia. The Nowruz holiday, festively celebrated in many countries around the vernal equinox, should also be considered given the increasing interest in Turkey’s eastern ski resorts from Iran and Azerbaijan.
